# DOSCATs: Double standards for protein quantification

**DOI:** 10.1038/srep45570

**Published:** 2017-04-03

**Authors:** Richard J. Bennett, Deborah M. Simpson, Stephen W. Holman, Sheila Ryan, Philip Brownridge, Claire E. Eyers, John Colyer, Robert J. Beynon

**Affiliations:** 1 Centre for Proteome Research, Institute of Integrative Biology, Biosciences Building, University of Liverpool, Liverpool L69 7ZB, UK; 2 Institute of Ageing and Chronic Disease, The Apex Building, 6 West Derby St., Liverpool L7 8TX, UK; 3 Badrilla Ltd. Leeds Innovation Centre, School of Biomedical Sciences, University of Leeds, Leeds LS2 9JT, UK

## Abstract

The two most common techniques for absolute protein quantification are based on either mass spectrometry (MS) or on immunochemical techniques, such as western blotting (WB). Western blotting is most often used for protein identification or relative quantification, but can also be deployed for absolute quantification if appropriate calibration standards are used. MS based techniques offer superior data quality and reproducibility, but WB offers greater sensitivity and accessibility to most researchers. It would be advantageous to apply both techniques for orthogonal quantification, but workflows rarely overlap. We describe DOSCATs (**DO**uble **S**tandard con**CAT**amers), novel calibration standards based on QconCAT technology, to unite these platforms. DOSCATs combine a series of epitope sequences concatenated with tryptic peptides in a single artificial protein to create internal tryptic peptide standards for MS as well as an intact protein bearing multiple linear epitopes. A DOSCAT protein was designed and constructed to quantify five proteins of the NF-κB pathway. For three target proteins, protein fold change and absolute copy per cell values measured by MS and WB were in excellent agreement. This demonstrates that DOSCATs can be used as multiplexed, dual purpose standards, readily deployed in a single workflow, supporting seamless quantitative transition from MS to WB.

Accurate quantification of proteins is of critical importance in cell biology, proteomics, clinical biomarker discovery and systems biology. Two very different approaches to quantification are routinely adopted; those based on mass spectrometry (MS) and those based on (semi) quantitative western blotting (sqWB). The two methods differ, both in the technical demands and in the complexity of the associated equipment, as well as the confidence in the quantitative data generated.

Mass spectrometric methods are considered to be the gold standard for targeted protein quantification^1^,^2^,^3^. However, capital investment and the expertise required in setting up and executing an MS assay means that it is less widely used than sqWB. For relative MS quantification, there is increasing application of label-free quantification based on the intrinsic signal intensity of individual peptides (derived from a digested protein) or of label-mediated quantification in which stable isotope labels are used to discriminate between two or more conditions, discriminated by the mass shift either at the level of the peptide ion or at the level of fragment ions generated within the mass spectrometer. Label based quantification methods are commonly used in conjunction with a targeted MS approach known as selected reaction monitoring (SRM). SRM utilises triple quadrupole mass spectrometers to perform two levels of mass selection, at the level of both precursor and product ion, giving much improved selectivity and sensitivity over global, ‘discovery’ proteomic approaches. Semi-quantitative western blotting is, by contrast, readily delivered with a small investment in equipment, and in most laboratories, requires extended sequences of manual processing steps (although there are instrumentation developments that automate the method). Although considered a semi-quantitative technique for relative quantification of signal intensity, sqWB is commonly used to draw quantitative conclusions despite the lack of calibration standards, rigorous (and standardised) methodology, and consistent data analysis^4^,^5^. However, direct comparison of sqWB results between groups is problematic as the data (effectively, the intensity of an antibody–reactive band that is generated by different chemistries and measured using different imaging devices) are dimensionless and highly variable (inter-assay) despite high levels of care and skill by the researcher. This limitation is likely to have contributed to the lack of reproducibility in pre-clinical data, which has a high cost in terms of wasted effort and delayed progress^6^,^7^. Many papers that report sqWB data do not include exhaustive data that defines the specificity of the antibody-antigen interaction, linearity of response or evidence that the immunoreactive band is the target antigen. Indeed, it is common practice in publication of sqWB results to crop western blot images to the region of interest, thus obscuring other regions of cross-reactivity. In sqWB, quantification is usually relative, where one condition is compared with a second, ideally run on the same gel and developed as a single blot.

For absolute quantification, calibration standards based on stable isotope labelled proteins or peptides (for MS) or epitope bearing proteins (for WB) are required. Isotope standards for MS, based on relatively short tryptic peptides, are not suitable for western blot quantification, such that MS-based and WB workflows rarely overlap. Ideally, there would be readily deployable technologies to converge techniques, raising standards in quantitative output. There is a continuing need for appropriate calibration standards in the western blot workflow, thereby creating genuinely quantitative western blotting (QWB). Further, it would be ideal if calibration standards were capable of deployment across both MS and QWB workflows. This crossover would allow for validation of the orthogonal techniques and comparison of data between the two most common quantitative techniques. Moreover, QWB could be used to improve characterisation of research antibodies (sensitivity, dynamic range, limit of detection, consistency of batches, specificity), which is currently problematic^8^,^9^ and a major factor in the irreproducibility of pre-clinical science^5^. Indeed, the use of orthogonal techniques such as MS to validate antibody specificity has been proposed by an international working group of scientists^10^.

It is easy to understand why western blotting is the preferred method (Pubmed searches reveal approximately 5,000 citations using the terms ‘SRM or MRM’, compared to over 250,000 using the terms ‘western blot’ or ‘western blotting’), as it is readily deployable in most laboratories and does not require access to either the specialist instrumentation or the cognate expertise that is needed to develop an MS-based method. However, there is considerable scope for studies that compare and explore the comparative performance of the two approaches. Indeed, it is a common experience with MS-based proteomics studies that reviewers request ‘validation’, implicitly meaning western blotting, without any evidence for the ability of both methods to deliver comparable data sets^1^. In pursuit of reliable approaches to quantitative western blotting, there is merit in comparison of such approaches with an orthogonal method, such as those based on mass spectrometry. Further, QWB methods give added information about electrophoretic mobility (crudely indicative of protein mass), information that is absent from ‘bottom-up’ proteomics.

We have previously designed and employed artificial proteins to solve problems in MS-based analyses, including QconCATs^11^ for absolute quantification and QCAL^12^, QCAL-IM^13^ and RePLiCal^14^ as universal standards for MS-based techniques. In particular, we developed QconCAT proteins to facilitate multiplexed protein quantification by MS^11^,^15^. QconCATs are artificial genes encoding proteins that are concatenations of (usually tryptic) peptides from multiple proteins (typically up to 25) that act as ‘quantotypic’ standards when the calibrator protein is expressed in bacteria and labelled with stable isotope amino acids^16^. After co-digestion of the analyte (in a biological sample) and the QconCAT, the resultant pairs of unlabelled (analyte) and labelled (standard) peptides can be analysed by mass spectrometry, permitting accurate quantification of the analyte protein abundance.

Here, we extend QconCAT principles to design DOSCATs ‘**DO**uble **S**tandard con**CAT**amers’, dual-purpose calibration standards for MS-based quantification and/or QWB, also designed *de novo* and expressed heterologously in *Escherichia coli*. DOSCATs concatenate epitope sequences from one or more proteins recognised by multiple antibodies. They also embed quantotypic peptides (Q-peptides) for MS quantification of the same proteins (Fig. 1) and thus act as a single multiplexed standard that can be used for MS-based or QWB quantification. For added flexibility, the epitopes can be interspersed with restricted specificity endoproteolytic sequences, which permit generation of quantification standards of optimal mobility in sized-based or charge-based separation platforms. The DOSCAT design includes a His-tag for purification and glufibrinopeptide B (Glu-Fib) sequence for quantification of the standard^17^ and contains at least two Q-peptides for each target protein, chosen according to well-defined criteria^16^,^18^. It is axiomatic that the epitopes can only be used if they are relatively short peptide sequences, either used as an immunogen or identified as the linear sequence that is recognised by a monoclonal antibody. The aim of this work is to demonstrate that DOSCATs can be used as a calibration standard across both SRM and QWB workflows to deliver equivalent quantitative results.

## Results and Discussion

### DOSCAT design, expression and purification

For DOSCATs, epitopes for a small panel of antibodies are inserted into the protein in addition to Q-peptides so that the standard can be deployed across both MS and QWB workflows. A minimum of two Q-peptides were chosen for each target protein based on a well-defined process^16^,^18^. The data repositories PeptideAtlas (http://www.peptideatlas.org/) and Global Proteome Machine (http://www.thegpm.org/) were initially consulted and peptides identified. Where peptides could not be identified using this approach (for IκBα and RelB), peptides were selected on the basis of a computational prediction of their quantotypic propensity based on physio-chemical properties and predicted observability (PeptideSieve score (http://tools.proteomecenter.org/wiki/index.php?title=Software:PeptideSieve)). For all selected Q-peptides, uniqueness in the proteome was confirmed by BLAST searches. Each peptide was supplemented with natural flanking sequences of 3 amino acids in length as a strategy to improve quantitative accuracy by equalising digestion efficiency between standard and analyte^19^ (Supplementary Table 1). For p65 and IκBβ, selected Q-peptides were adjacent in the protein sequence so flanking regions were not possible nor required. Epitopes included in the DOSCAT sequence were chosen based on the immunogen sequence supplied by the manufacturer (Supplementary Table 2). Where the precise immunogen sequence was disclosed by the manufacturer, this was used in the DOSCAT - this was the case for IκBβ and IκBε. For some antibodies, only the specific residue around which the epitope was centred was disclosed and in these instances, up to 25 amino acids flanking each side of the central residue in the protein sequence were used to ensure inclusion of the epitope in the DOSCAT sequence. This was required for the epitopes for p65, RelB and IκBα.

**Figure 2:**
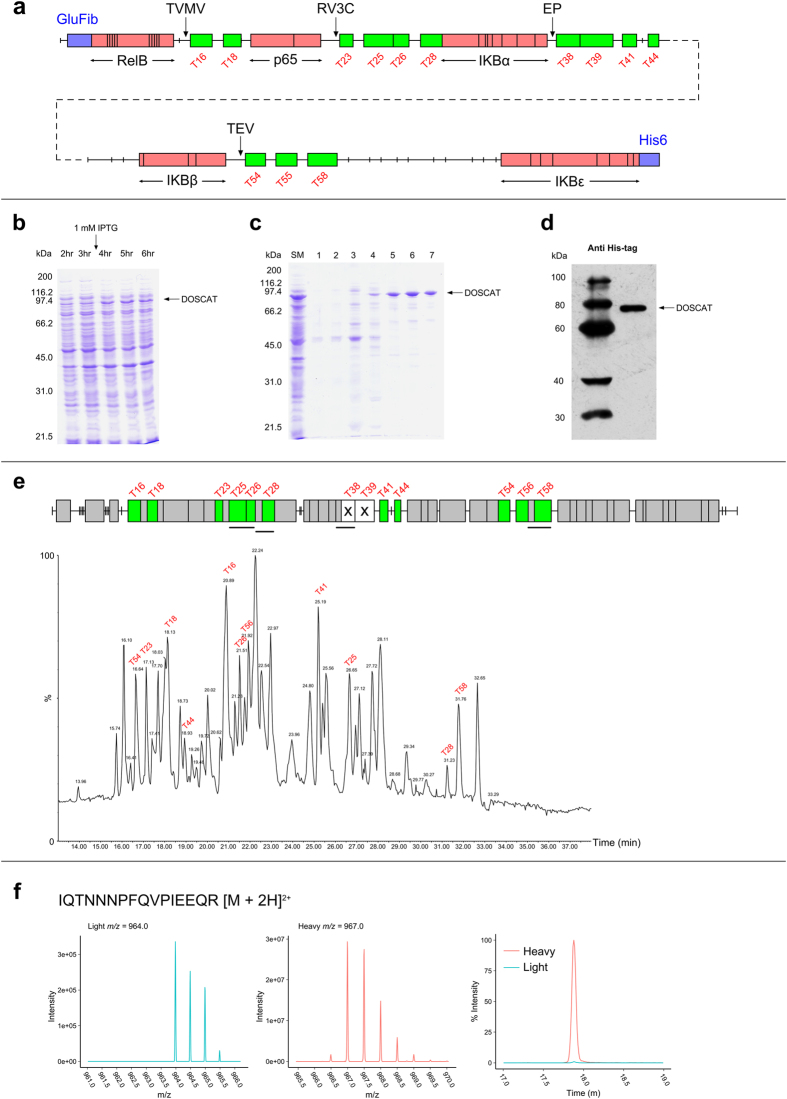
Design of a DOSCAT for quantification of members of the NFκB pathway. (**a**) Protein map of the NF-κB DOSCAT. Green boxes represent quantotypic peptides, red boxes define the extent of antibody binding epitopes and purple boxes the glu-fibrinopeptide B calibration peptide at the N-terminus and the His6 purification tag at the C-terminus. Arrows indicate the location of cleavage sites for each of the specific proteases (TVMV, tobacco vein mottling virus protease; RV3C, human rhinovirus 3 C protease; EP, enteropeptidase; TEV, tobacco etch virus protease. SDS-PAGE analysis of (**b**) *E. coli* culture time points 2–6 hrs after inoculation, with expression induced by IPTG after 3 hrs and (**c**) pre-purification starting material (SM) alongside elution fractions 1–7 from His-Trap column using an elution gradient 0–100% elution buffer over 20 min. (**d**) Western blot analysis of 50 ng purified DOSCAT using an anti His-tag antibody. (**e**) DOSCAT peptide map highlighting Q-peptides identified (green) and not identified (white with cross) by MS/MS, alongside the MS1 total ion chromatogram signifying the elution profile of each Q-peptide. (**f**) Mass spectra and SRM chromatogram for a representative Q-peptide demonstrating high stable isotope labelling efficiency.

To introduce the possibility of manipulation of mobility in QWB, several restricted specificity endopeptidase sites were included in the DOSCAT design. These sites were selected based on the primary sequence specificity and the commercial availability of the protease. Four endopeptidases were used (Supplementary Table 3) and for each, the target sequence was unique to a single site in the DOSCAT and was absent from any of the target proteins. Cleavage sites were inserted into the sequence between epitopes so that proteolysis would generate two fragments that could be detected by different antibodies and which would have a different electrophoretic mobility to the parent DOSCAT, permitting a mobility shift to prevent overlap between standard and analyte. Additional amino acids were added at the N-terminus to provide an initiator methionine and a sacrificial N-terminal region as well as glufibrinopeptide B peptide (EGVNDNEEGFFSAR) for MS-based quantification of the standard^3^. A hexahistidine tag was added at the C-terminus to allow affinity purification. Once the protein sequence was designed (Fig. 2a) and the codon optimised gene was synthesised, the DOSCAT was expressed successfully in *E.coli* in minimal medium (Fig. 2b), accumulating in inclusion bodies, and after solubilisation in a chaotrope was readily purified by Ni-NTA affinity chromatography (Fig. 2c). The protein sequence and optimised gene sequence for the DOSCAT are included in Supplementary Information.

### Validation and performance in QWB and SRM

After purification, the DOSCAT was homogenous on SDS-PAGE and western blotting using an anti His-tag antibody (Fig. 2d) confirmed that there was no proteolysis of the protein during expression or purification. Moreover, western blotting using p65 and RelB antibodies (the epitopes to which are at the N-terminal end), demonstrated there was no degradation or fraying at the N-terminus of the protein (Supplementary Fig. 1). After expression and purification, the DOSCAT was subject to tryptic digestion and the peptides were analysed by LC-MS/MS on a Synapt G2. This confirmed that the protein sequence was correct with 83% sequence coverage and 11 of the 13 nominated Q-peptides being identified by LC-MS/MS (Fig. 2e). The peptide DAGADLDKPEPTCGR was not identified due to a miscleavage event at the N terminus. Only the miscleaved peptide, containing the preceding residues DDDDK, the cleavage site for enteropeptidase, was detected. It is known that acidic residues around the scissile bond can inhibit proteolysis^20^,^21^; therefore, it is likely that the sequence context around the peptide was the reason for the miscleavage. Care should be taken in placing Q-peptides next to such residue sequences when designing future iterations of DOSCATs and this observation casts doubt on the utility of enteropeptidase as a restricted specificity proteinase in this application. Additionally, the peptide SPLHLAVEAQAADVLELLLR was not detected either by database searching or in the raw data. This was further investigated by running the same DOSCAT digest on a QExactive Orbitrap instrument. The peptide was detected by database searching and in the raw data, but with a signal intensity ~0.1% of the base peak intensity. This lack of detectability on two separate instrument platforms confirm poor ionisation efficiency or fragmentation of the peptide after ionisation. When the DOSCAT was expressed in minimal media containing [^13^C_6_]Lys and [^13^C_6_]Arg as the sole source of these amino acids, complete labelling (>99%) of the protein was confirmed by the examination of MS1 data and extracted ion chromatograms (Fig. 2f).

**Figure 3:**
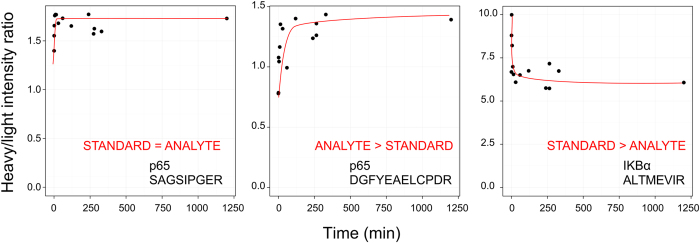
Time course to complete digestion of DOSCAT and sample. Stable isotope labelled DOSCAT and SK-N-AS lysate was co-digested with samples taken at a series of time points and analysed by SRM-MS. The ratio of heavy (DOSCAT) and light (endogenous) peptides was calculated at each time point. Displayed are exemplar plots for the three types of observed digestion behaviour: peptide release in standard and analyte digestion being equal and rapid (STANDARD = ANALYTE), faster in analyte (ANALYTE > STANDARD), or faster in standard (STANDARD > ANALYTE). Red lines indicate general trend of H:L ratio over time.

For a DOSCAT to be used for the accurate quantification of target proteins, complete and equivalent digestion of standard and analyte is crucial. Although it is possible that peptides may be released more quickly from either standard or analyte, the rate of digestion in both should reach a plateau before analysis^22^. To ascertain whether there was complete release of Q-peptides from the DOSCAT, the standard was spiked into SK-NA-S cell lysate and the digestion mixture was sampled at regular intervals. Rates of excision of standard and analyte peptides varied (for exemplars, see Fig. 3), but in all instances the proteolysis attained a stable plateau before the end of the overnight incubation period (Supplementary Fig. 2). Differences in the rate of digestion between standard and analyte are thus rendered moot by the simple expedient of establishing conditions that allow the reactions to proceed to completion.

**Figure 4:**
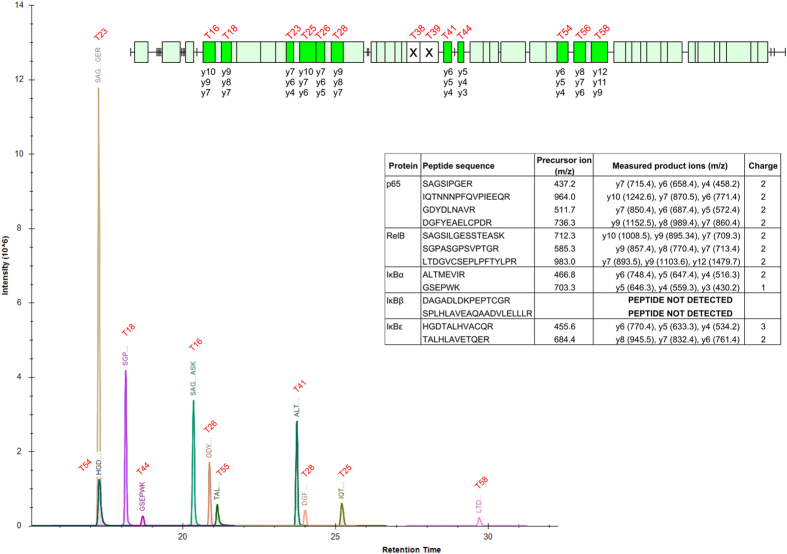
Summary of scheduled SRM-MS assays for Q-peptides contained in DOSCAT. Extracted ion chromatogram from SRM-MS analysis of 1 fmol digested DOSCAT loaded onto the column, detailing the peak intensity and retention time for all Q-peptides for which scheduled SRM-MS assays were built. Inset: DOSCAT peptide map illustrates all Q-peptides for which SRM-MS assays were designed (bright green boxes) out of all observed peptides (pale green boxes) alongside product ions monitored for each peptide. Two Q-peptides were not detectable by SRM-MS (white box with cross). Inset table contains a detailed list of transitions for each peptide.

To define the transition of the SRM assays, a tryptic digest of the DOSCAT was analysed by LC-MS/MS using a Q-TOF mass spectrometer. For each peptide, MS/MS fragmentation data were used to identify optimal transitions for each peptide (Supplementary Fig. 3). These transitions were then used to build a scheduled SRM profile and a programme of timed transitions resulted in cleanly isolated peaks specific for all but two of the Q-peptides (Fig. 4). To determine performance in a complex matrix, DOSCAT was spiked in SK-NA-S lysate at concentrations over a 1000-fold range and analysed by SRM. The signal linearity varied for each peptide, with some peptides exhibiting a linear relationship of the entire measured range. Moreover, the limits of detection (LOD) (based on a signal to noise ratio of 3) also differed for each peptide (Supplementary Fig. 4) with an average LOD of 100 amol. Based on the number of cell equivalents loaded onto the column, this corresponds to about 10,000 copies per cell.

**Figure 5:**
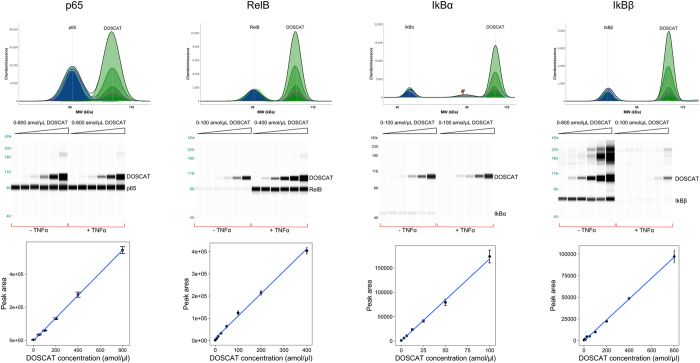
Quantitative western blots. Representative Simple Western data for 0.4 μg/μl SK-NA-S cell lysate ± TNFα stimulation spiked with a dilution series of DOSCAT and probed with antibodies for each target protein, shown in electropherogram view (top) and gel view (middle). Bottom: Calibration curves generated by DOSCAT for each protein. Data presented as mean ± standard error (n = 6).

To validate the DOSCAT for western blotting, dilution series of the standard protein were loaded onto the capillary western blotting system and were detected independently by each of the five commercial antibodies (Fig. 5). Sensitivities varied for different antibodies, ranging from 5 amol to 1.5 fmol in each lane, defined as the lower limit of quantification. Based on the number of cell equivalents that were loaded into the capillaries, this equates to about 1000 copies per cell for the lowest limit of detection. IκBε could not be quantified using this methodology as the antibody did not detect the epitope in DOSCAT. However, when used against SK-NA-S cell lysate in both capillary and classic western blotting, the antibody detected a band at ~90 kDa (data not shown), very different from the expected molecular weight (53 kDa). We conclude that the antibody was unreliable and it was not used in further experiments.

Although this DOSCAT met many of the criteria of a dual-purpose standard, the critical test of such a standard is in the quantification of endogenous proteins. Specifically, would both QWB and MS assays yield acceptable quantification, and secondarily, would the presence of a complex background such as a cell homogenate impair the ability to use the standard? Further, the migration of the DOSCAT in the capillary QWB system had the potential to lead to interference with the signal for the endogenous analyte. To explore a realistic scenario for deployment of the artificial dual standard, DOSCAT was spiked into protein extracts of unstimulated and TNFα-stimulated SK-N-AS cells. The same cell lysates, spiked with the DOSCAT standard, were used for MS or QWB quantification, the difference being that for QWB, the spiked lysates were analysed without further treatment, whereas for MS quantification, the mixture was reduced, alkylated and proteolysed with trypsin. The DOSCAT was accurately quantified against an unlabelled Glu-Fib standard^17^,^23^ before subsequent SRM-MS and QWB analysis.

QWB was performed on the same samples in parallel with SRM-MS. The DOSCAT was serially diluted in SK-N-AS cell lysates to create an internal calibration series for each lysate. DOSCAT/lysate mixtures were analysed by automated western blotting system with antibodies that were specific and had been validated for each of the target proteins. The DOSCAT was designed to migrate at an apparent molecular weight (Mr) that differed from endogenous target proteins, allowing separation of signal between the exogenous DOSCAT standard and endogenous analytes (Fig. 5). In practice, we observed some baseline interference at very high loadings of standards when the DOSCAT and analyte were similar in mobility, leading to less accurate quantification of peak area (Supplementary Fig. 5). This was readily resolved by restricting the standard data points to exclude the highest internal DOSCAT concentrations (Supplementary Fig. 6). This adjustment was applied for all target proteins, regardless of similarity in mobility between analyte and standard. Moreover, analyte levels were always within the linear calibration region of the standard curve. The DOSCAT signals were used to construct calibration curves that were linear in all cases (r^2^ > 0.98, exemplar in Fig. 5, all data in Supplementary Fig. 7).

For SRM-MS, the extracted ion chromatograms for unlabelled analyte and the labelled Q-peptide (released from DOSCAT) were used to calculate the amount of analyte present, as copy number per cell (cpc) values (using the calculation described in Materials and Methods). Protein level quantification values were calculated by averaging peptide values across technical replicates and in turn, taking the mean of the values obtained for different peptides. More than one peptide per protein was detected for p65 and RelB; for p65 the peptide cpc values were in good agreement but for RelB, SGPASGPSVPTGR gave lower cpc values than the other peptides (Supplementary Fig. 8). This may be due to the proximity of the endogenous peptide to the N-terminus or the presence of a previously unknown post translational modification on the peptide, and such the peptide was not included in calculating the final cpc value. Despite nominating peptides based on experimental evidence, neither IκBβ, IκBε nor the the IκBα peptide GSEPWK could be quantified by SRM-MS due to a combination of miscleavage potential in the standard, poorly performing peptides and the intrinsic low abundance of the target proteins^16^.

**Figure 6:**
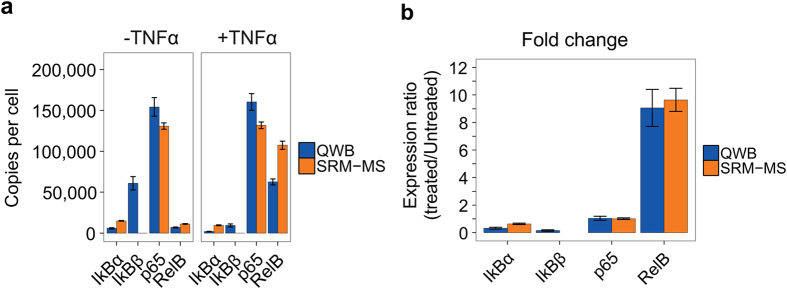
Target protein quantification. (**a**) Comparison of absolute quantification values for each target protein as obtained by SRM-MS and QWB. Quantification of IκBβ using SRM was not possible. Data presented as mean ± standard error (n = 3). (**b**) Relative fold change of proteins in TNFα treated and untreated SK-NA-S cells as measured by quantitative western blotting (QWB) and selected reaction monitoring (SRM-MS). Data are presented as mean ± standard error (n = 3).

Two measures were used to compare the performance of the orthogonal analytical approaches. First, each method yielded absolute quantification values in copies per cell. Secondly, the changes in protein abundance after stimulation by TNFα could be compared. The utility of DOSCAT was demonstrated by quantification of five target proteins in the NF-κB pathway using both quantitative platforms. Exposure of cells to the cytokine TNFα significantly increases the levels of endogenous RelB and elicits degradation of the inhibitor proteins of NF-κB, IκBs^24^. DOSCAT standardised quantification by SRM-MS or QWB were consonant with this expectation, giving confidence to the method. Although only a relatively small number of proteins were quantified in this study, agreement in copy numbers between the two techniques were improved compared to other studies that have compared MS and WB quantification^3^,^25^,^26^. In terms of both copies per cell and relative fold change, quantitative values generated by QWB and SRM-MS were in agreement for the 3 out of 5 proteins where comparison was possible (Fig. 6a,b). Moreover, both techniques demonstrated high precision with a mean % CV across both techniques of 14% (biological replicates, n = 3). This is a clear demonstration that careful design of standards, coupled with appropriate technology and experimental design can converge these orthogonal methodologies.

Accurate protein quantification by orthogonal techniques is desirable to increase accuracy and robustness of quantitative data, especially important in systems analysis of specific pathways or biomarker validation. Quantification by two methodologies in parallel in a single experiment is difficult, though, due to differences in calibration standards. To address this problem we have proposed DOSCATs as a single calibration standard to support quantification by both QWB and SRM-MS assays across a single workflow. In some instances, QWB allowed quantification whereas SRM-MS did not, due to lack of peptide detection at low protein abundance and poor peptide ionisation. This further emphasises the value of using orthogonal techniques to quantify proteins across a wide dynamic range, or small proteins where options for Q-peptides are restricted. In our hands both quantitative platforms demonstrated an equivalently high level of precision using DOSCAT, which was within the range typically reported for SRM and QWB (using Simple Western technology) assays^3^,^27^,^28^. Using ‘classic’ western blotting, it would be anticipated that reproducibility would be lower due to the greater number of manual handling steps. With rigorous experimental design and well validated antibodies^5^,^29^, there is no reason why similarly equivalent quantitative data could be obtained using DOSCAT as a calibration standard.

**Figure 7:**
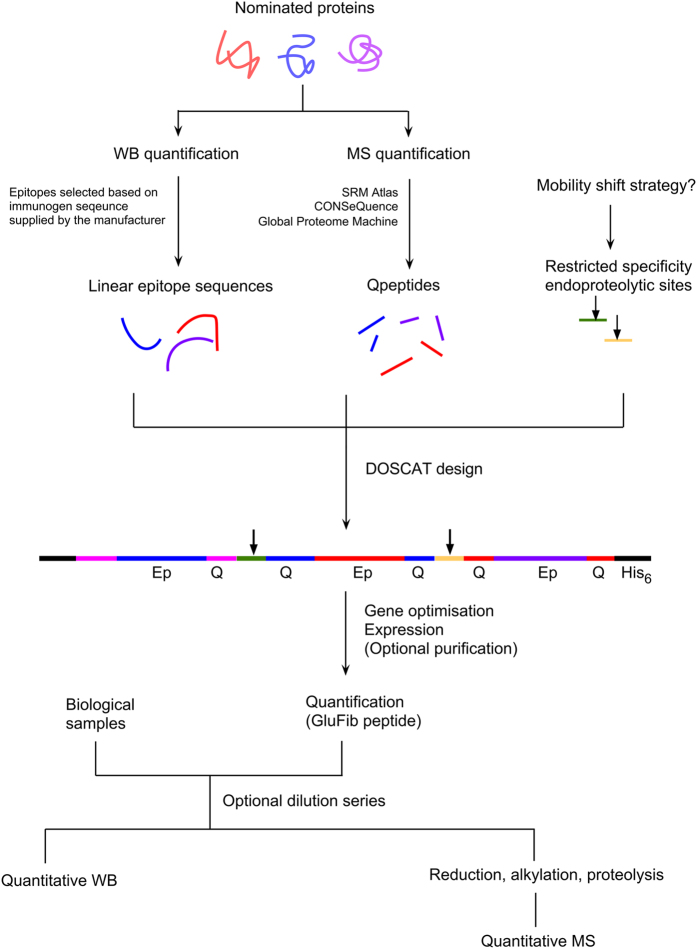
Overall DOSCAT workflow. The deployment of DOSCAT quantification can be resolved into three phases; design, expression and assay development. This workflow summarises the major steps.

Based on well-established QconCAT technology for which much of the route to deployment is well characterised^23^,^30^ (~900 citations citing QconCATs are recorded in Google Scholar as of December 2016), the DOSCAT workflow is simple to implement (a generic workflow is presented in Fig. 7). Since the initial publication in 2005^11^, over 200 QconCATs have been utilised in a range of quantitative studies. QconCATs differ to natural proteins in amino acid composition^17^ and almost always accumulate in inclusion bodies. Expression in inclusion bodies can provide a useful pre-purification step but can also create problems of insolubility in long-term storage post purification – the use of MS-compatible detergents such as RapiGest *SF* and a low concentration of a reducing agent can minimise such problems^17^. The NF-κB DOSCAT expression is resonant with these previous observations, and we would expect future DOSCATs to follow this trend. In terms of design, selection criteria for Q-peptides are well documented^16^,^18^ and can equally be applied to DOSCATs. Of course, the availability of antibodies that are cross-reactive to known linear epitopes is a limiting factor when constructing a DOSCAT. However, a search for the term ‘synthetic peptide’ in the CiteAb (https://www.citeab.com/) database revealed over half a million antibodies raised to linear synthetic peptides. Thus, in an environment that was permissive to collaboration with antibody manufacturers, or where custom antibodies are made in-house, these peptide sequences could be included in DOSCATs. We encourage antibody manufacturers and suppliers to release the peptide sequences used for antibody generation as a contribution to reproducible research but also, to ease the construction of standards such as DOSCATs. If restricted proteolytic sites are built into the DOSCAT sequence, there is also an element of adaptability in the workflow so that fragments of a predictable electrophoretic mobility are produced upon incubation with specific proteases. This not only allows for additional flexibility if the DOSCAT standard migrates at a similar mobility to endogenous proteins but the ability to create standards containing epitopes separable by limited proteolysis can assist in multiplexed protein detection in a single lane or capillary of a western blot.

DOSCATs offer a new calibration tool for protein quantification by both SRM-MS and QWB and unite two disparate workflows by a single calibration standard yielding equivalent quantification. Western blotting is one of the most widely used research techniques practised by the majority of cell biologists, despite previous limitations in delivering quantitative data. The DOSCAT approach has the potential to enhance the rigour of QWB that is more readily applied after MS validation, to generate reliable quantitative information particularly relevant for systems biology studies and contribute to the desired increase in reproducibility of biological research.

## Materials and Methods

### Materials

Antibodies used in this study are detailed in Supplementary Table 2.

### DOSCAT design

DOSCATs are based on QconCAT design principles, in which quantotypic peptides are concatenated *in silico* to create an artificial protein^11^. For DOSCATs, epitopes for multiple antibodies and specific protease cleavage sites are inserted into the protein as well as quantotypic peptides. A minimum of two quantotypic peptides were chosen for each target protein for SRM. The data repositories PeptideAtlas and Global Proteome Machine were initially consulted and suitable peptides were identified based on well-defined criteria^16^,^18^. Natural flanking sequences consisting of 3 amino acids from the endogenous protein were added to each end of every peptide^31^. This strategy has been shown to improve quantitative accuracy by equalising digestion efficiency between standard and analyte. Where peptides could not be identified (notably, IκBα and RelB) other quantification peptides for DOSCAT were selected based on their PeptideSieve score and predicted observability.

Epitopes included in the DOSCAT sequence were chosen based on the immunogen sequence supplied by the manufacturer (Supplementary Table 2). If the full immunogen sequence was disclosed by the manufacturer, this was used in the DOSCAT, as was the case for IκBβ and IκBε. For some antibodies only the residue around which the epitope was centred was disclosed. In these cases up to 25 amino acids either side of the central residue in the protein sequence were used in the DOSCAT sequence, specifically for p65, RelB and IκBα. Proteases were selected based on the specificity of their cleavage motif and commercial availability. Cleavage sites were inserted into the sequence between epitopes so that proteolysis would result in two fragments that could be detected by different antibodies. Additional amino acids were inserted at the N-terminus to provide an initiator methionine as well as a glufibrinopeptide B (EGVNDNEEGFFSAR) for standard quantification. A hexahistidine tag was added at the C-terminus to enable purification. Protein and DNA sequences as FASTA files are included in Supplementary Information.

### Expression and purification of stable isotope labelled DOSCAT

The gene for the DOSCAT protein was optimised for expression in *Escherichia coli*, synthesised and ligated into a pET21a plasmid vector (Eurofins Genomics, Ebersberg, Germany). The plasmid was transformed into competent BL21 DE3 *E.coli* cells, which were cultured in minimal media containing ^13^C_6_ labelled arginine and lysine as previously described^23^. DOSCAT expression was induced by Isopropyl β-D-1-thiogalactopyranoside (1 mM) when the culture OD at 600 nm reached 0.6. *E.coli* cells were separated from culture media by centrifuging at 3500 × *g*, 15 min, 4°C. Cell pellets from 50 mL culture were resuspended in 2.5 mL sonication buffer (50 mM NaPO_4_, 25 U/mL Benzonase Nuclease (Novagen), 1× Complete EDTA-free protease inhibitor tablet (Roche), pH 8.0). Cells were sonicated on ice using 10 sec pulses at 30% amplitude delivered from a 3 mm probe of a Sonics Vibra Cell™ (Jencons Scientific Ltd, UK) until 130 joules was reached.

The *E.coli* lysate was centrifuged at 6000*** **g*, 8 min, 4°C to separate insoluble and soluble fractions. DOSCAT was present in the insoluble inclusion body pellets, which were solubilised by incubating for 30 min in 4 mL 20 mM NaPO_4_, 0.5 M NaCl, 10 mM imidazole, 6 M guanidine hydrochloride, pH 7.4. Inclusion body samples were filtered using a 1.20 μm syringe filter (Milliex GP, Merck Millipore, UK) before purification of the His-tagged DOSCAT on a 1 mL His-trap HP column (equilibrated in the solubilisation buffer, above) using the ÅKTA start system (GE Healthcare). Bound proteins were eluted by applying a linear gradient of 0–100% elution buffer (20 mM NaPO_4_, 0.5 M NaCl, 0.5 M imidazole, 6 M guanidine hydrochloride, pH 7.4) over 20 min at a flow rate of 1 mL/min. Eluted fractions containing DOSCAT were pooled and dialysed against 50 mM ammonium bicarbonate (NH_4_HCO_3_), 1 mM DTT, pH 8.5. RapiGest *SF* (Waters, UK) was added to the storage buffer at a final concentration of 0.1% (w/v) in order to reduce DOSCAT adsorption to plastic surfaces. DOSCAT solution was aliquoted and stored in low bind tubes (Corning, USA) at −20°C.

### Harvesting, counting and sonication of cell lysates

SK-N-AS cells between passages 7–14 were grown to 80% confluency in 75 cm^2^ flasks in Minimum Essential Media (37°C, 5% CO_2_/humidity). In each set of experiments a set of three cultures were stimulated with TNFα (10 ng/mL) for a period of 24 hrs. At the same time media was replaced on the unstimulated cells without added TNFα. To harvest cells, media was aspirated and adherent cells were washed three times with sterile PBS (5 mL). Cell dissociation buffer (1 mL) was added and the cells placed in the incubator at 37°C for 5 min. PBS (4 mL) was added to each flask and the contents of each flask transferred to individual 15 mL Falcon tubes. Cells were dispersed by repeated uptake and aspiration from a 5 mL pipette and 50 μL of cell suspension was removed and added to an equal volume of 0.4% (w/v) trypan blue in PBS. Immediately after mixing, dye suspension (10 μL) of cells was pipetted into each of the two chambers of a counting slide and cell numbers counted in a TC10 cell counter (Bio-RAD, Hemel Hempstead, UK). The counter gave a direct reading of the total cells/mL and the viable cells/mL of suspension. Cells were harvested by centrifugation at 160 *g* (Eppendorf, Cambridge, UK), supernatant removed and cell pellets stored at −20°C.

Cell pellets were re-suspended in 25 mM ammonium bicarbonate (not pH adjusted) at 100 μL/1 × 10^6^ cells and sonicated on ice using three 10 sec pulses at 30% amplitude delivered from a 3 mm probe of a Sonics Vibra Cell™. Benzonase nuclease (2.5 U/100 μL cell lysate) was added and the cell lysate was held on ice and not fractionated further.

### Protein determination

Protein was assayed using a modified Bradford assay. Cell lysate and DOSCAT were diluted 1:50 and 1:100 in Milli-Q (18 Ω) water and 100 μL of each sample added to a microtitre plate in duplicate followed by the addition of 200 μL protein assay reagent (Thermo Fisher Scientific, Cramlington, UK). The plate was read at 600 nm on a microplate reader (Multiscan) using Ascent software (Thermo Fisher Scientific) and protein concentrations interpolated from a BSA standard curve.

### DOSCAT and SK-N-AS cell lysate sample preparation

A known concentration of DOSCAT was spiked into SK-N-AS lysate and RapiGest *SF* added at a final concentration of 0.1% (w/v) to create a starting master mix, which was subsequently used for both SRM-MS and QWB workflows. A sample of the master mix was digested, accurate DOSCAT concentration determined using glufibrinopeptide B (Severn Biotech, >98% purity), and targeted protein quantification performed by SRM. The same master mix was then used for quantitative western blotting.

### In solution co-digestion

A volume of master mix equivalent to 70 μg total protein in cell lysate was processed through the tryptic digest protocol. Alongside the cell lysate/DOSCAT co-digest, a cell lysate only digest was performed in parallel for each biological replicate. The samples were made up to 160 μL by the addition of 25 mM ammonium bicarbonate and proteins were denatured by the addition of Rapigest (0.1% w/v) and heating at 80°C for 10 min. The sample was then reduced (addition of 10 μL 60 mM DTT and heating at 60°C for 10 min) and alkylated (addition of 180 mM iodoacetamide and incubation at RT for 30 min in the dark). In digests containing DOSCAT, 4.5 μL 500 fmol/μL glufibrinopeptide B was added. Mass spectrometry grade trypsin (Promega, USA) was reconstituted in 50 mM acetic acid to 0.2 μg/μL and 10 μL added to digests followed by incubation at 37°C for 4.5 hrs. At this stage an additional 2 μg trypsin was added and the sample incubated at 37°C overnight. The digestion was ended and RapiGest *SF* removed by acidification (1.5 μL trifluoroacetic acid followed by incubation at 37°C for 45 min). Digests were made up to 225 μL by the addition of acetonitrile:water (2:1) and precipitate (resultant from the breakdown of RapiGest *SF*) removed by centrifugation (13,000 *g*, 30 min, 4°C). Supernatant was removed from the precipitate pellet and carried forward for use as sample.

### Quantification of labelled DOSCAT using glufibrinopeptide B peptide standard

Samples were analysed using nanoAcquity UPLC™ system (Waters) coupled to a Xevo™ TQS triple quadrupole mass spectrometer (Waters) in SRM mode with Q1 and Q3 operating at unit mass resolution. Digested sample (1 μL) was loaded onto a trapping column (C18, 180 μm × 20 mm, Waters) using partial loop injection for 3 min at a flow rate of 5 μL/min with 0.1% (v/v) formic acid. The sample was resolved on the analytical column (nanoACQUITY UPLC HSS T3, C18, 75 μm × 150 mm × 1.8 μm column, Waters) using a gradient of 97% A (0.1% (v/v) formic acid) 3% B (99.9% (v/v) acetonitrile, 0.1% (v/v) formic acid) to 60% A 40% B over 30 min at a flow rate of 300 nL/min followed by washing with buffer B and re-equilibration. SRM analysis was performed using an electrospray voltage of 3000 V and a source temperature of 80°C. Glufibrinopeptide B was detected by measuring three transitions of both isotopic variants over the entire course of the chromatographic run. Quantification was performed by integrating extracted ion chromatograms in heavy and light channels and comparing the two.

### Quantification of peptides by SRM and data analysis

Quantification was performed on a Xevo™ TQS triple quadrupole mass spectrometer coupled to a nanoAcquity UPLC™ system (Waters) (using the parameters described above). Analysis was performed using the same settings as previously described in scheduled SRM mode in which 15 data points were acquired over a 30 sec chromatographic peak within a 4 min window. Collision energy was optimised for each peptide (Supplementary Table 1).

Samples were prepared so that 1 μg cell lysate with either 1 fmol or 0.1 fmol DOSCAT were analysed by SRM methodology. Samples were prepared by serial dilution of the master mix sample with the cell lysate only digest. Both isotopic variants of each target peptide were analysed by three transitions. The final transition list was divided in two runs to achieve a minimum dwell time of 30 msec with each sample being analysed with both transition lists.

Data were analysed by Skyline^32^ and absolute quantification values were calculated from the standard:analyte ratio and the known concentration of internal stable isotope labelled standard. Quantification as copies per cell was derived from knowledge of the number of cells loaded onto the column and the loading of the accurately quantified DOSCAT standard. For the IκBα peptide GSEPWK, neither analyte nor DOSCAT standard could be measured. This could be due to a lack of peptide fragmentation or poor chromatographic behaviour^16^. Despite having peptides chosen based on experimental evidence, neither IκBβ nor IκBε could be quantified by SRM-MS due to a combination of miscleavage potential in the standard, poorly performing peptides and the intrinsic low abundance of the target proteins. All raw data files were deposited in PASSEL with accession number PASS00979.

### Quantitative western blotting and data analysis

A volume of master mix was serially diluted in SK-N-AS cell lysate (of equal SK-N-AS cell lysate concentration to that of the master mix) containing no DOSCAT so as to create a series of samples which contained a range of DOSCAT concentrations. A six-point DOSCAT calibration curve and six technical replicates of the SK-N-AS cell lysate were analysed by automated western blotting (Wes, ProteinSimple, CA) for each protein.

Samples were prepared for Simple Western analysis according to the ProteinSimple user manual. Samples were mixed at a 4:1 ratio with a 5× master mix (ProteinSimple) containing SDS, 40 mM DTT and fluorescent molecular weight standards and heated at 95°C for 5 min. Samples plus biotinylated molecular weight standards (ProteinSimple) were loaded along with blocking solution, wash buffers, primary antibodies, horseradish-peroxidase conjugated secondary antibodies and chemiluminescent substrate into a plate prefilled with stacking and separation matrices. Antibodies were used at concentrations as detailed in Supplementary Table 2. Fully automated western blotting was performed using the Wes system; proteins were separated by electrophoreses at 375 V for 25 min, immobilised to the capillary by proprietary UV crosslinking and incubated with primary and secondary antibodies for 30 min each. Chemiluminescent signal was captured by a charge-coupled device camera and the resulting image was analysed by Compass software (ProteinSimple). All Compass data files are included in Supplementary Data. The signal intensity was converted to moles μg^−1^ using the DOSCAT calibration curve, and subsequently converted to copies per cell.

## Additional Information

**How to cite this article:** Bennett, R. J. *et al*. DOSCATs: Double standards for protein quantification. *Sci. Rep.* **7**, 45570; doi: 10.1038/srep45570 (2017).

**Publisher's note:** Springer Nature remains neutral with regard to jurisdictional claims in published maps and institutional affiliations.

## Supplementary Material

Supplementary Information

## Figures and Tables

**Figure 1:**
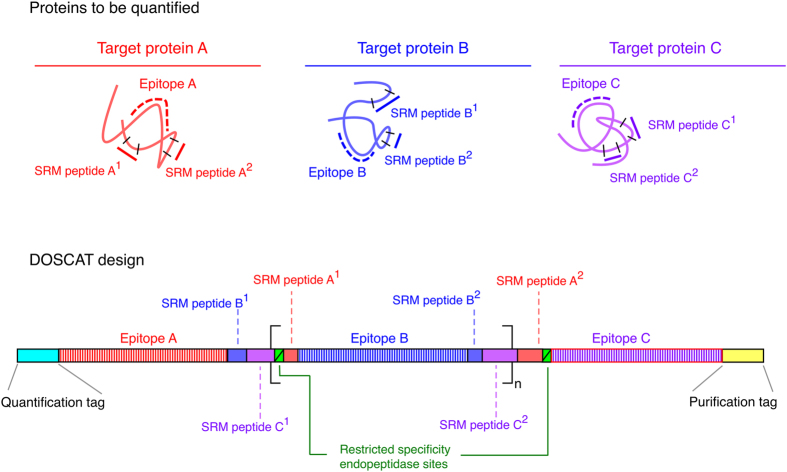
Principle of DOSCAT design. Peptides for MS-based quantification and antibody epitopes are selected for each target protein and sequences concatenated into a single artificial protein (DOSCAT). Tag sequences for quantification and purification of DOSCAT are inserted at each terminus. Restricted specificity endopeptidase sites are interspersed throughout the sequence so as to permit generation of quantification standards of optimal mobility during sized-based separation analysis.
