# Supplementary material for: DOSCATs: Double standards for protein quantification
Source: Sci Rep. 2017 Apr 3;7:45570. doi: 10.1038/srep45570 (PMC5377311; doi:10.1038/srep45570)
Supplement: Supplementary Information [file srep45570-s1.pdf]

DOSCATs: Double standards for protein quantification

Richard J. Bennett[1], Deborah M. Simpson[1], Stephen W. Holman[1], Sheila Ryan[2], Philip Brownridge[1], Claire Eyers[1], John Colyer[3,*], Robert J. Beynon[1,*]

1: Centre for Proteome Research, Institute of Integrative Biology, Biosciences Building, University of Liverpool, Liverpool L69 7ZB

2: Institute of Ageing and Chronic Disease, The Apex Building, 6 West Derby St., Liverpool L7 8TX

3: Badrilla Ltd. Leeds Innovation Centre, Leeds LS2 9DF, UK; School of Biomedical Sciences, University of Leeds, Leeds LS2 9JT

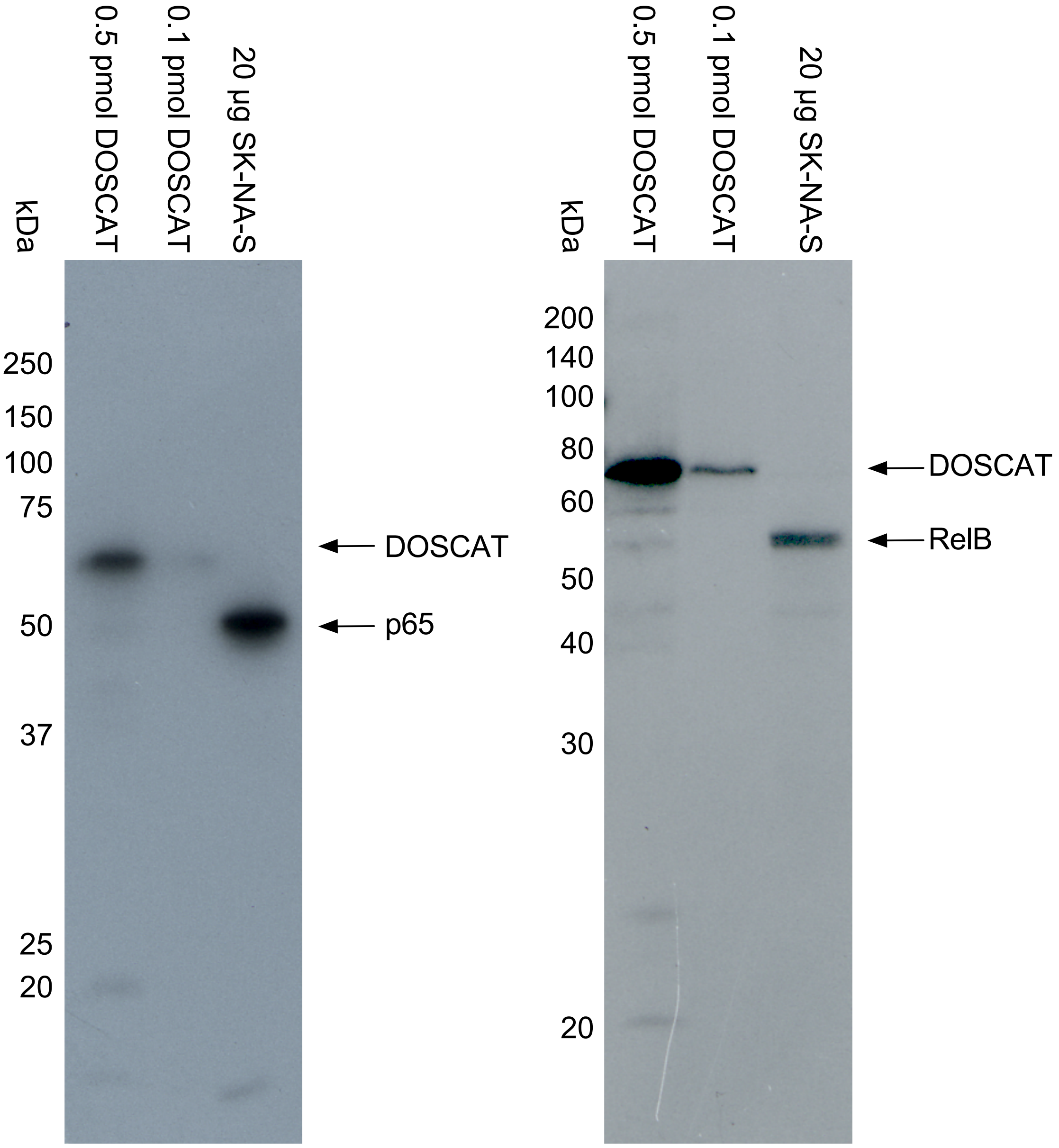

Supplementary Figure 1

Traditional gel-based western blot of DOSCAT and SK-NA-S cell lysate probed with anti p65 and RelB antibodies.

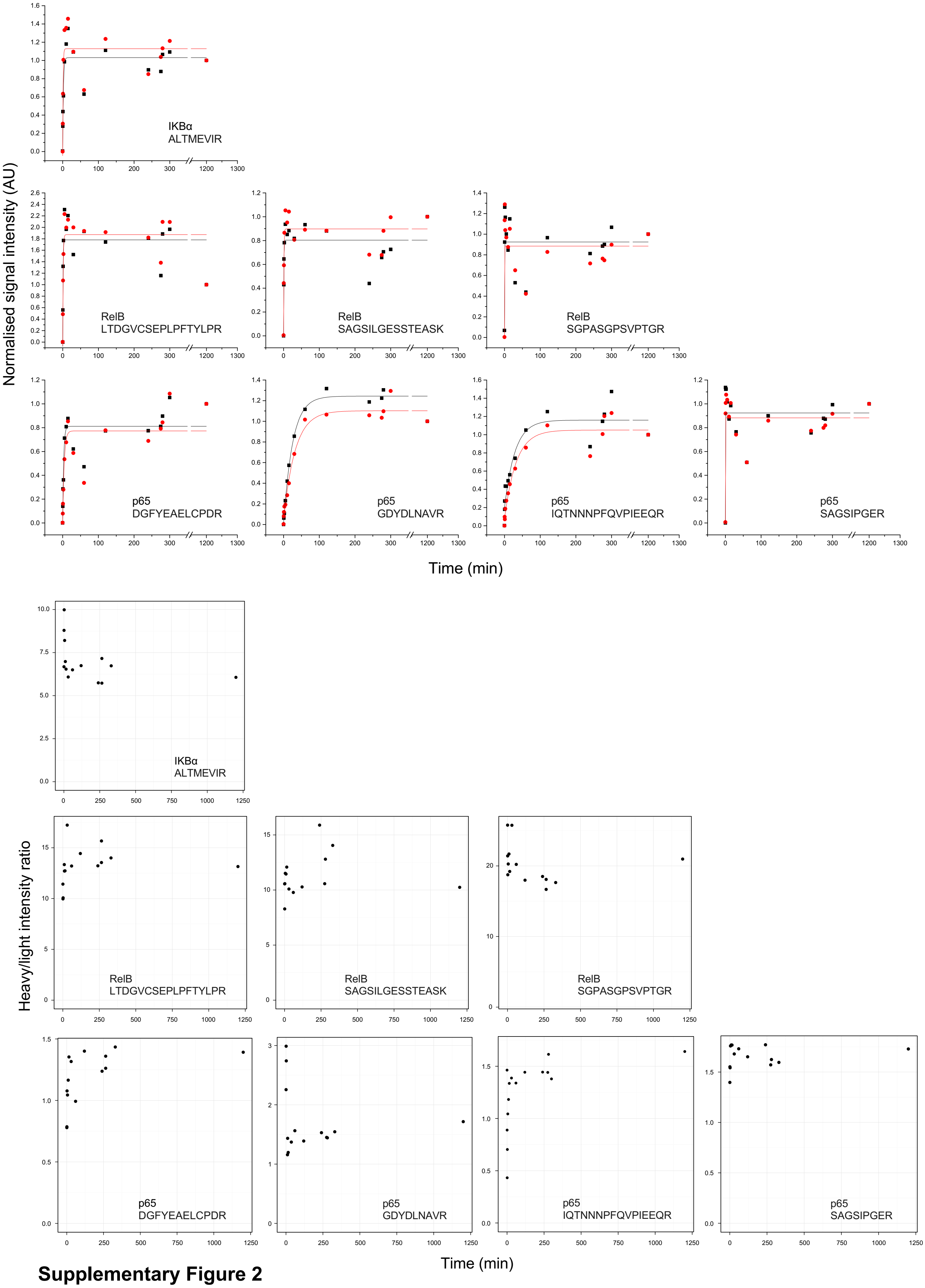

Supplementary Figure 2

Time course analysis showing the release of each peptide used for quantification during tryptic digestion. Stable isotope labelled DOSCAT and SK-N-AS lysate was co-digested, with samples taken at a series of time points and analysed by SRM-MS. Displayed are signal intensities normalised to the final time point for heavy (DOSCAT) and light (endogenous) peptides (top), and the ratio of heavy to light peptide at each time point (bottom).

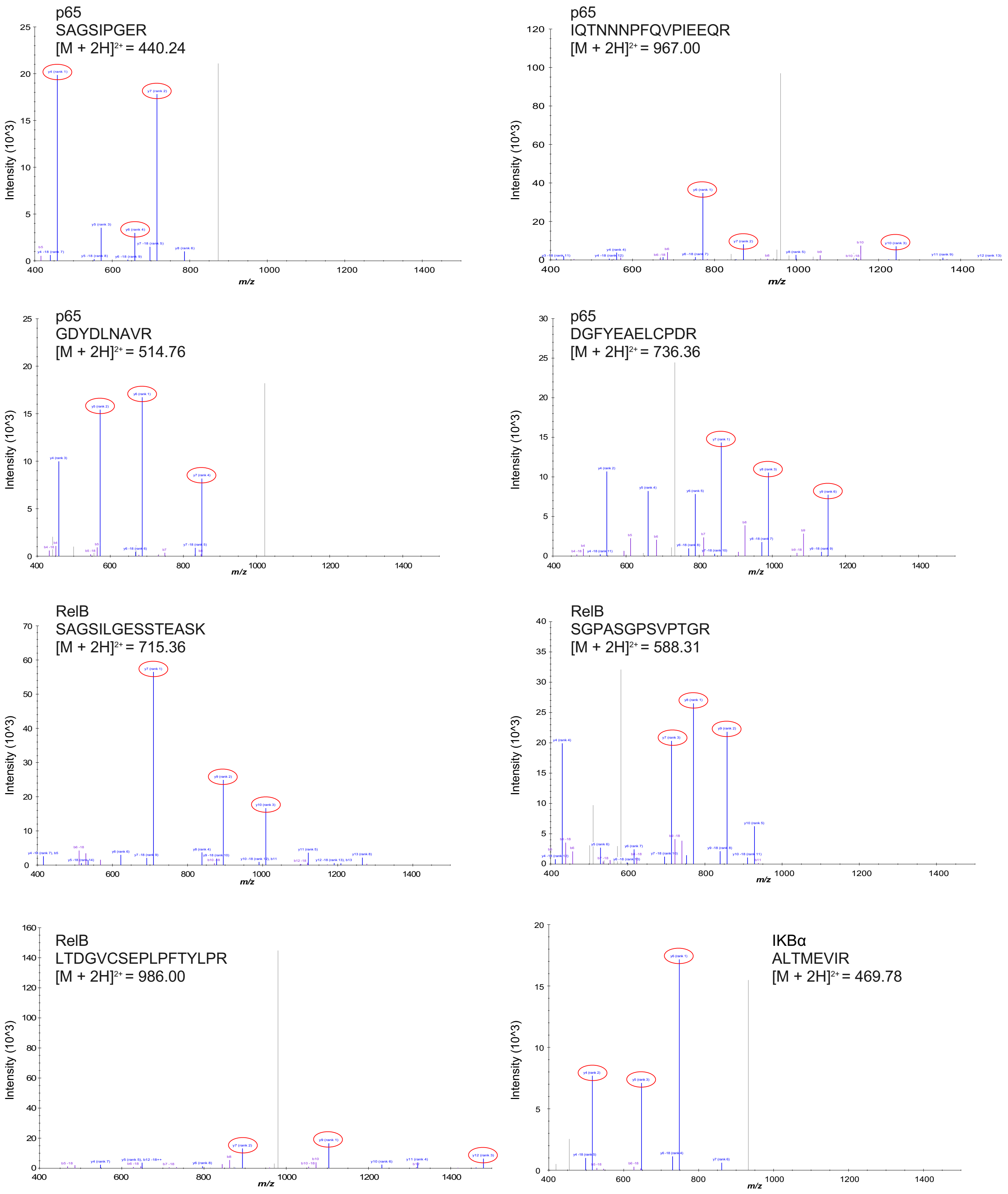

Supplementary Figure 3

MS/MS spectra for each Q-peptide used for quantification generated by analysis of a trpytic digest of DOSCAT2 by a Q-TOF instrument. Observed product ions chosen for analysis by SRM-MS are highlighted in red.

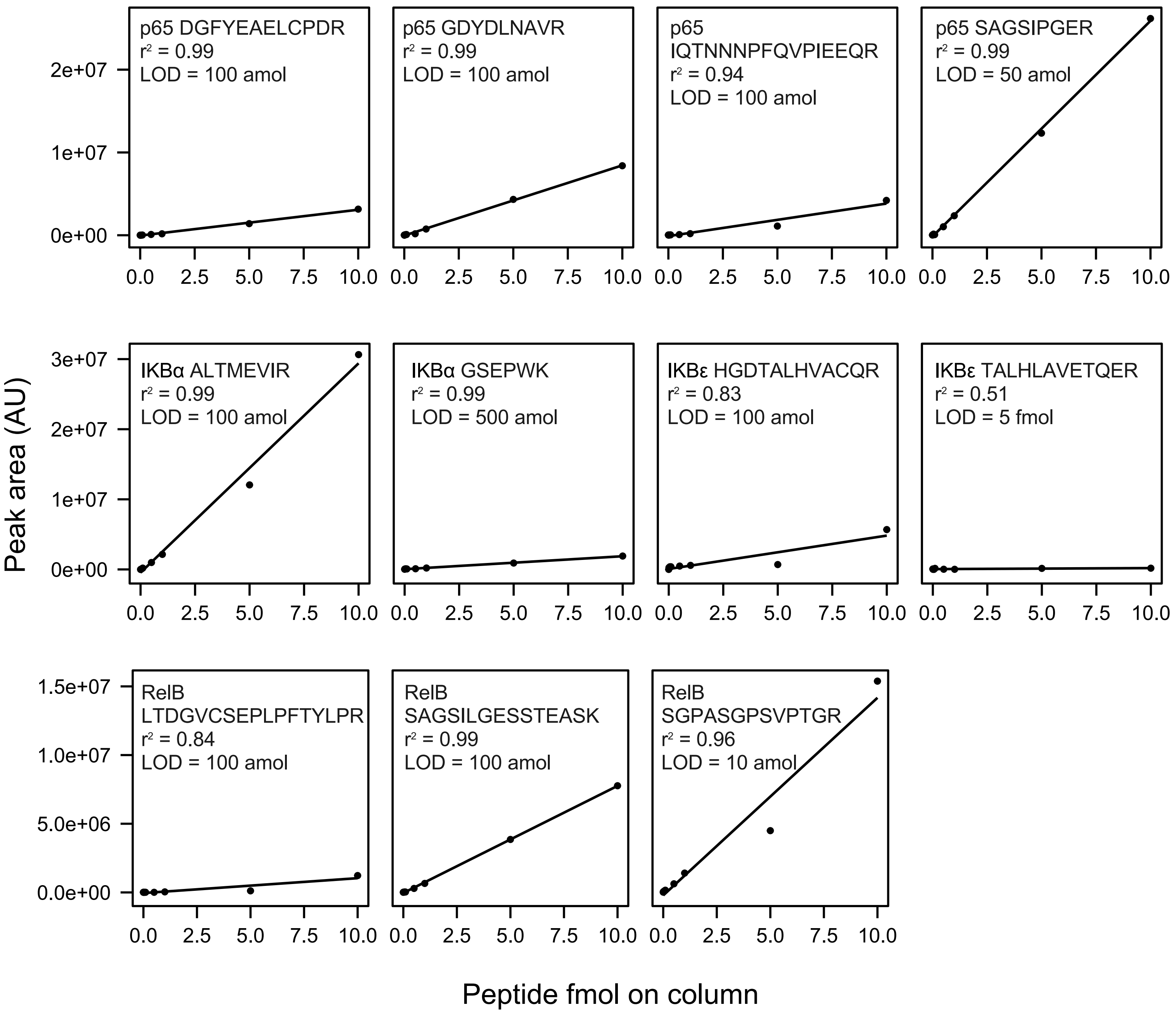

Supplementary Figure 4

Standard curve for each Q-peptide in SRM-MS assays generated by co-digesting DOSCAT in SK-NA-S cell lysate and serially diluting in SK-NA-S cell lysate digest. Also displayed are measures of limit of detection (where signal:noise = 2) and linearity of response from 10 amol to 10 fmol material on column.

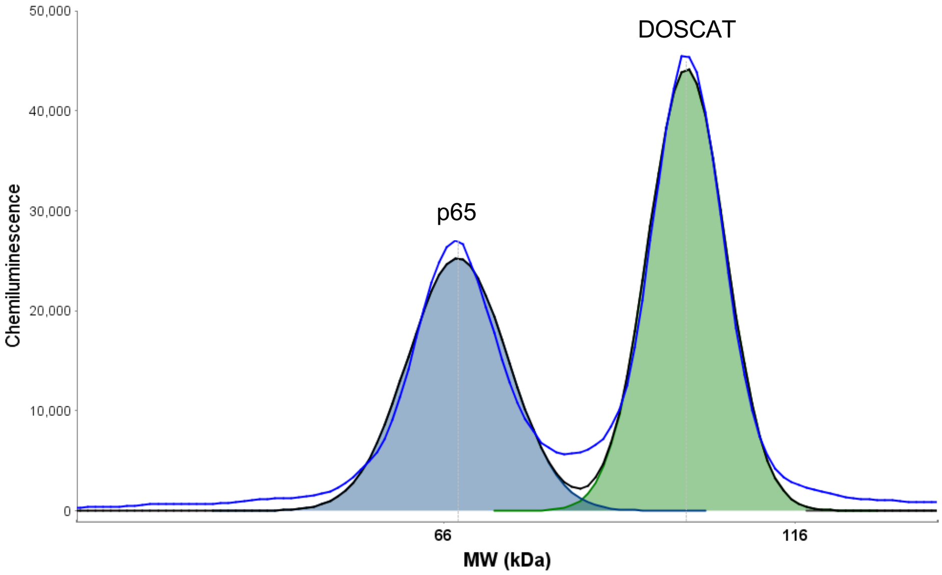

Supplementary Figure 5

Electropherogram trace generated by automated western blotting for endogenous p65 and DOSCAT standard highlighting the overlap in peak fitting between the analyte and standard.

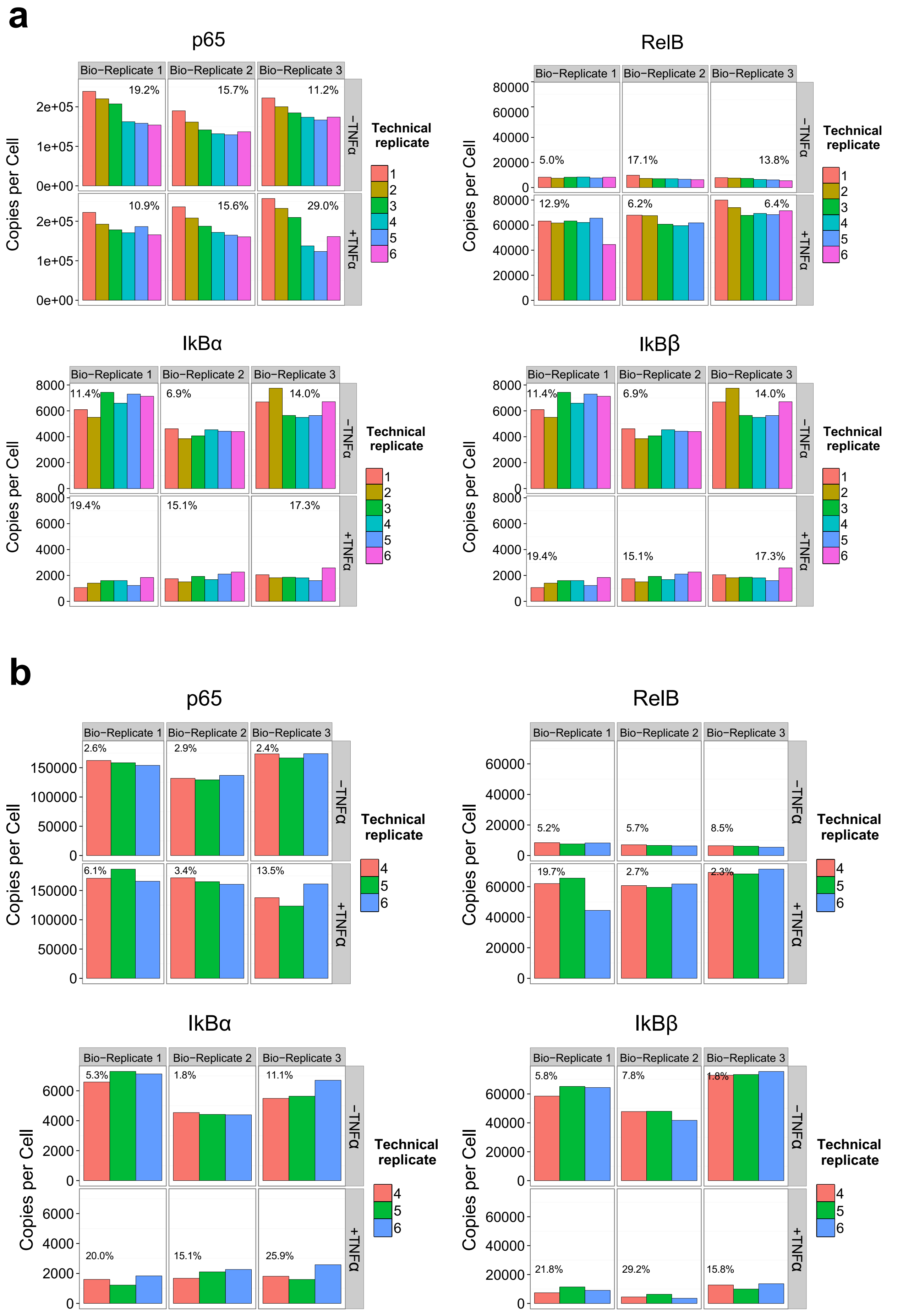

Supplementary Figure 6

Quantification values determined by quantitative western blotting factored by biological replicate and treatment type (±TNFα) for a) all six technical replicates; b) the three technical replicates paired with the lowest spike-in DOSCAT concentrations. Percentage value in each panel represents % CV across the technical replicates

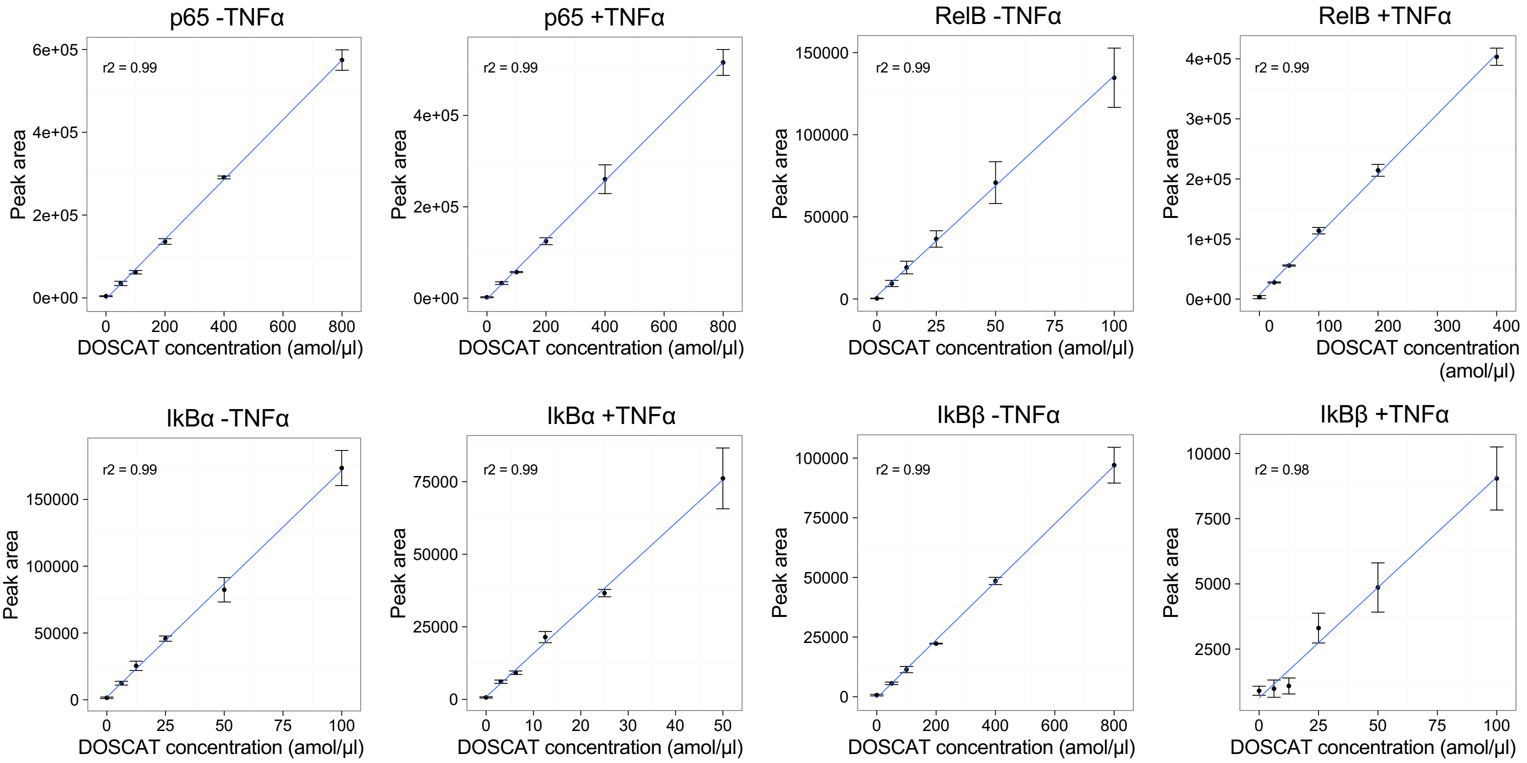

Supplementary Figure 7

Calibration curves created using four target antibodies against DOSCAT standard spiked into SK-NAS lysate +/- TNFα. DOSCAT signal at each point averaged across three biological replicates for each treatment type. Error bars represent standard error.

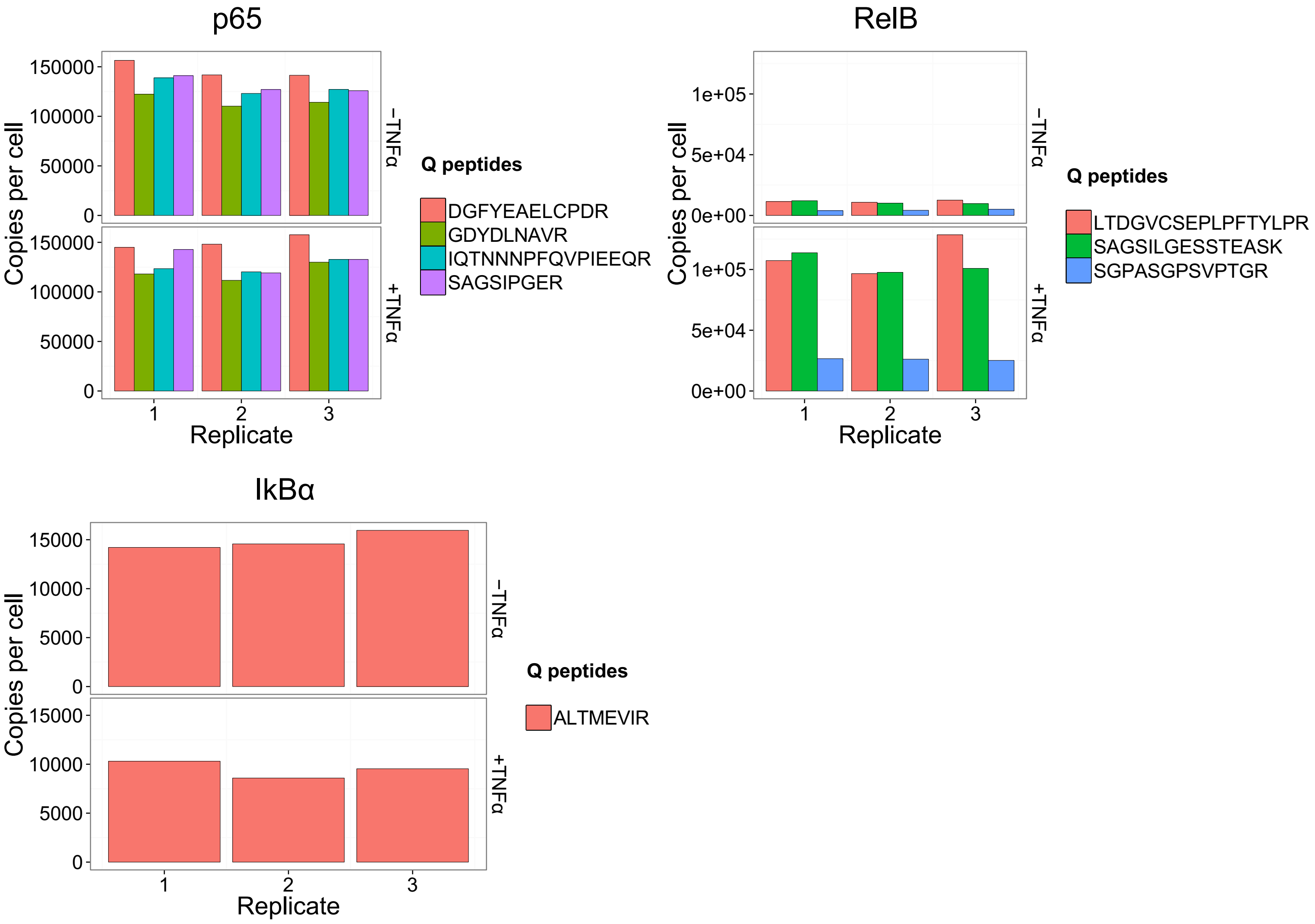

Supplementary Figure 8

Quantification values in copies per cell for each detected Q-peptide as determined by SRM-MS.

Supplementary Table 1 | SRM transitions, collision energies and dwell times for the analysis of each target peptide.Natural flanking sequences of peptides are shown in red.

Protein	Peptide sequence	Precursor ion (m/z)	Measured product ions (m/z)	Charge	Collision energy (eV)	Dwell time (msec)
p65	EGRSAGSIPGERSTD	437.2	y7 (715.4), y6 (658.4), y4 (458.2)	2	13	38
	SQRIQTNNNPFQVPIEEQR	964.0	y10 (1242.6), y7 (870.5), y6 (771.4)	2	37	30
	GDYDLNAVRLCF	511.7	y7 (850.4), y6 (687.4), y5 (572.4)	2	16	38
	DCRDGFYEAELCPDRCIH	736.3	y9 (1152.5), y8 (989.4), y7 (860.4)	2	27	30
RelB	EGRSAGSILGESSTEASKTLP	712.3	y10 (1008.5), y9 (895.34), y7 (709.3)	2	26	30
	MLRSGPASGPSVPTGRAMV	585.3	y9 (857.4), y8 (770.4), y7 (713.4)	2	20	30
	LQRLTDGVCSEPLPFTYLPRDHD	983.0	y7 (893.5), y9 (1103.6), y12 (1479.7)	2	38	163
IκBα	EEKALTMEVIRQVK	466.8	y6 (748.4), y5 (647.4), y4 (516.3)	2	25	30
	VPRGSEPWKQQL	703.3	y5 (646.3), y4 (559.3), y3 (430.2)	1	30	48
IκBβ	DAGADLDKPEPTCGR		PEPTIDE NOT DETECTED			
	SPLHLAVEAQAADVLELLLRAGA		PEPTIDE NOT DETECTED			
IκBε	QDRHGDTALHVACQRQHL	455.6	y6 (770.4), y5 (633.3), y4 (534.2)	3	9	38
	SGKTALHLAVETQERQLV	684.4	y8 (945.5), y7 (832.4), y6 (761.4)	2	24	163

Supplementary Table 2 | Details of antibodies used in western blotting and the epitopes built into DOSCAT sequence.

**Protein**	**Manufacturer**	**ID**	**Lot**	**Clonality**	**Species**	**Antibody dilution**	**Immunogen**	**Epitope in DOSCAT**
**p65**	Cell Signalling Technology	#8242S	4	Monoclonal	Rabbit	1:1000	Centred around Glu 498 in human p65 (Q04206)	**L478** NQGIPVAPHTTEPMLMEYPEITRLV TGAQRPDPAPAP **L518**
**RelB**	Cell Signalling Technology	#4954	5	Polyclonal	Rabbit	1:500	Centred around Ser424 in human RelB (Q01201)	**D401** HDSYGVDKKRKRGMPDVLGELNSS DPHGIESKRRKKKPAILD **H444**
**IκBα**	Cell Signalling Technology	#9242	10	Polyclonal	Rabbit	1:100	Centred around Arg29 in human IκBα (P25963)	**M1** FQAAERPQEWAMEGPRDGLKKERL LDDRHDSGLDSMKDEEYEQMVKEL QEI **R53**
**IκBβ**	Bethyl Laboratories Inc.	A301-828A	1	Polyclonal	Rabbit	1:100	Between residue 306 and 356 in human IκBβ (Q15653)	**E306** KSGPCSSSSDSDSGDEGDEYDDIVVH SSRSQTRLPPTPASKPLPDDPRP **V356**
**IκBε**	Abcam	ab51147	GR974 07-5	Polyclonal	Rabbit	1:50	IESLR synthetic peptide – corresponds to residues 158 to 163 in human IκBε (O00221)	**Q154** YDSGIESLRSLRS **L168**

Supplementary Table 3 | Restricted specificity endoproteases used and their cleavage sequence inserted into DSOCAT.

Protease	Manufacturer	Cleavage sequence
Tobacco etch virus (TEV)	Invitrogen, USA	ENLYFQG
Human rhinovirus 3C (RV3C)	GE Healthcare , UK	LEVLFQGP
Tobacco vein mottling virus (TVMV)	Biomol, Germany	TVRFQS
Enteropeptidase	Novagen, UK	DDDDK

DNA sequence and translation of DOSCAT 2

```
1        M  G  T  R  E  G  V  N  D  N  E  E  G  F  F  S  A  R  D  H  D  S  Y  G  V  D  K  K  R  K  R  G  M
     CATATGGGCACGCGTGAAGGCGTCAACGATAACGAAGAGGGCTTCTTCTCTGCGCGTGACCATGACTCCTATGGCGTCGACAAGAAACGCAAACGTGGGA 100

      P  D  V  L  G  E  L  N  S  S  D  P  H  G  I  E  S  K  R  R  K  K  K  P  A  I  L  D  H  R  T  V  R
101  TGCCGGATGTCCTGGGTGAACTGAATTCCAGTGATCCACACGGCATAGAGTCAAAGCGTCGCAAAAAGAAACCAGCGATTCTCGACCATCGCACGGTTCG 200

      F  Q  S  E  G  R  S  A  G  S  I  L  G  E  S  S  T  E  A  S  K  T  L  P  M  L  R  S  G  P  A  S  G
201  GTTTCAGTCGGAAGGTCGTAGTGCGGGCAGCATCCTCGGCGAATCCAGCACGGAAGCGTCTAAAACCCTGCCGATGTTGCGTTCTGGTCCAGCCAGCGGT 300

       P  S  V  P  T  G  R  A  M  V  F  R  L  N  Q  G  I  P  V  A  P  H  T  T  E  P  M  L  M  E  Y  P  E  A
301  CCAAGTGTACCTACAGGACGCGCGATGGTGTTTCGCTTGAATCAGGGGATTCCGGTTGCACCGCACACCACAGAACCAATGCTGATGGAATACCCCGAAG 400

      I  T  R  L  V  T  G  A  Q  R  P  P  D  P  A  P  A  P  L  R  L  E  V  L  F  Q  G  P  E  G  R  S  A
401  CGATTACTCGCTTGGTTACAGGTGCTCAGCGCCCTCCAGATCCAGCGCCTGCACCACTTAGGCTGGAAGTTTTGTTCCAAGGCCCGGAAGGCAGATCAGC 500

      G  S  I  P  G  E  R  S  T  D  S  Q  R  I  Q  T  N  N  N  P  F  Q  V  P  I  E  E  Q  R  G  D  Y  D
501  TGGGTCAATTCCAGGGGAAAGGAGCACCGATAGCCAGCGAATCCAGACGAACAACAATCCGTTTCAGGTGCCGATTGAGGAACAACGTGGAGACTACGAC 600

      L  N  A  V  R  L  C  F  D  C  R  D  G  F  Y  E  A  E  L  C  P  D  R  C  I  H  M  F  Q  A  A  E  R  P
601  CTGAACGCAGTCCGCTTGTGCTTCGACTGTCGTGATGGCTTCTACGAAGCGGAGTTATGCCCTGACCGGTGTATCCACATGTTTCAGGCAGCGGAACGTC 700

      Q  E  W  A  M  E  G  P  R  D  G  L  K  K  E  R  L  L  D  D  R  H  D  S  G  L  D  S  M  K  D  E  E
701  CCCAAGAGTGGGCAATGGAAGGTCCACGGGATGGCTTAAAGAAAGAGCGGCTGCTAGACGATCGCCATGATAGTGGCCTAGACTCGATGAAAGACGAGGA 800

      Y  E  Q  M  V  K  E  L  Q  E  I  R  D  D  D  D  K  D  A  G  A  D  L  D  K  P  E  P  T  C  G  R  S
801  ATACGAGCAGATGGTCAAAGAGCTACAGGAGATCCGTGACGACGATGACAAAGACGCCGGTGCGGACCTGGATAAACCGGAACCCACTTGCGGTAGAAGC 900

      P  L  H  L  A  V  E  A  Q  A  A  D  V  L  E  L  L  L  R  A  G  A  E  E  K  A  L  T  M  E  V  I  R  Q
901  CCGTTGCATCTGGCTGTAGAAGCTCAAGCGGCTGATGTGCTCGAATTGCTACTGCGTGCAGGCGCAGAAGAGAAAGCCCTGACGATGGAAGTCATTCGCC 1000

      V  K  V  P  R  G  S  E  P  W  K  Q  Q  L  E  G  R  S  A  G  S  I  P  G  E  H  S  T  D  N  N  R  T
1001 AAGTGAAAGTTCCCAGGGGATCTGAGCCGTGGAAACAGCAACTTGAAGGTCGTTCGGCAGGTAGCATCCCGGGAGAACATAGCACCGACAATAACCGCAC 1100

      Y  P  L  L  R  V  G  A  D  P  A  L  L  D  R  E  K  S  G  P  C  S  S  S  S  D  S  D  S  G  D  E  G
1101 GTATCCGCTCCTTAGAGTGGGCGCAGATCCTGCTTTGCTCGATCGTGAGAAAAGTGGGCCGTGCTCCAGCTCATCTGATAGTGATAGCGGCGACGAAGGC 1200
```

```
       D  E  Y  D  D  I  V  V  H  S  S  R  S  Q  T  R  L  P  P  T  P  A  S  K  P  L  P  D  D  P  R  P  V  K
1201 GACGAGTATGATGATATTGTGGTTCATTCGTCCCGTTCCCAGACTCGGTTACCACCTACCCCAGCCTCTAAACCTCTACCCGACGATCCGAGGCCTGTGA 1300

        E  N  L  Y  F  Q  G  Q  D  R  H  G  D  T  A  L  H  V  A  C  Q  R  Q  H  L  S  G  K  T  A  L  H  L
1301 AAGAGAACCTGTACTTCCAGGGTCAGGATCGTCACGGTGATACCGCCTTACATGTAGCCTGTCAGCGTCAACACCTGTCTGGGAAAACTGCGCTTCATCT 1400

       A  V  E  T  Q  E  R  G  L  V  L  Q  R  L  T  D  G  V  C  S  E  P  L  P  F  T  Y  L  P  R  D  H  D
1401 GGCCGTGGAAACTCAGGAACGCGGGTTAGTACTCCAGCGTCTAACCGATGGCGTATGTAGCGAACCGCTGCCGTTTACGTATCTGCCTCGTGATCACGAT 1500

       T  P  R  S  G  N  T  N  P  L  S  S  F  S  T  R  T  L  P  L  Y  K  L  L  E  I  P  D  P  D  K  N  W  A
1501 ACCCCACGTTCGGGTAACACGAATCCGCTTAGCTCGTTTAGCACCCGTACCCTGCCGCTATACAAGCTTCTCGAGATCCCAGATCCCGATAAGAACTGGG 1600

       P  V  K  T  T  S  Q  A  H  S  L  P  L  S  P  A  S  T  R  E  L  V  N  M  R  N  D  L  Y  Q  T  P  L
1601 CGCCGGTGAAAACGACTTCGCAGGCCCATAGCCTGCCTCTATCGCCGGCTAGCACACGCGAACTCGTGAATATGCGCAATGACCTGTATCAGACCCCTCT 1700

       H  L  A  V  I  T  K  Q  E  D  N  A  R  L  F  G  L  A  Q  R  S  A  R  A  S  G  P  S  D  G  S  P  Q
1701 GCACCTTGCGGTGATCACCAAACAGGAAGATAACGCACGCCTGTTTGGACTTGCGCAACGAAGTGCGAGGGCGAGTGGTCCTTCAGATGGAAGTCCACAA 1800

       P  C  T  H  P  P  G  P  V  K  E  P  Q  E  K  E  D  A  D  G  E  R  A  D  S  T  Y  G  S  S  S  L  T  Y
1801 CCGTGCACCCATCCGCCTGGCCCTGTTAAGGAACCGCAAGAAAAGGAGGATGCCGATGGCGAACGCGCAGATTCTACCTACGGGTCATCGAGCCTGACCT 1900

       T  L  S  L  L  G  G  P  E  A  E  D  P  A  P  R  L  P  L  P  H  S  R  Q  Y  D  S  G  I  E  S  L  R
1901 ATACGCTGTCCCTGTTAGGTGGTCCTGAAGCCGAAGATCCTGCACCGCGTCTGCCTTTACCGCACTCACGCCAGTATGATAGCGGGATTGAATCCCTCCG 2000

       S  L  R  S  L  R  A  G  A  G  A  A  G  H  H  H  H  H  H  *  *  G  S
2001 CTCTCTGAGAAGCTTACGAGCTGGTGCCGGTGCGGCTGGCCACCATCACCATCACCACTAATAAGGATCC 2070
```
